# The coordinated management of ribosome and translation during injury and regeneration

**DOI:** 10.3389/fcell.2023.1186638

**Published:** 2023-06-22

**Authors:** Thanh Nguyen, Jason C. Mills, Charles J. Cho

**Affiliations:** ^1^ Department of Medicine, Baylor College of Medicine, Houston, TX, United States; ^2^ Department of Pathology and Immunology, Baylor College of Medicine, Houston, TX, United States; ^3^ Department of Molecular and Cellular Biology, Baylor College of Medicine, Houston, TX, United States

**Keywords:** ribosome, translation, injury, regeneration, paligenosis

## Abstract

Diverse acute and chronic injuries induce damage responses in the gastrointestinal (GI) system, and numerous cell types in the gastrointestinal tract demonstrate remarkable resilience, adaptability, and regenerative capacity in response to stress. Metaplasias, such as columnar and secretory cell metaplasia, are well-known adaptations that these cells make, the majority of which are epidemiologically associated with an elevated cancer risk. On a number of fronts, it is now being investigated how cells respond to injury at the tissue level, where diverse cell types that differ in proliferation capacity and differentiation state cooperate and compete with one another to participate in regeneration. In addition, the cascades or series of molecular responses that cells show are just beginning to be understood. Notably, the ribosome, a ribonucleoprotein complex that is essential for translation on the endoplasmic reticulum (ER) and in the cytoplasm, is recognized as the central organelle during this process. The highly regulated management of ribosomes as key translational machinery, and their platform, rough endoplasmic reticulum, are not only essential for maintaining differentiated cell identity, but also for achieving successful cell regeneration after injury. This review will cover in depth how ribosomes, the endoplasmic reticulum, and translation are regulated and managed in response to injury (e.g., paligenosis), as well as why this is essential for the proper adaptation of a cell to stress. For this, we will first discuss how multiple gastrointestinal organs respond to stress through metaplasia. Next, we will cover how ribosomes are generated, maintained, and degraded, in addition to the factors that govern translation. Finally, we will investigate how ribosomes and translation machinery are dynamically regulated in response to injury. Our increased understanding of this overlooked cell fate decision mechanism will facilitate the discovery of novel therapeutic targets for gastrointestinal tract tumors, focusing on ribosomes and translation machinery.

## 1 Chronic inflammation, metaplasia, and carcinogenesis in the GI tract

The gastrointestinal (GI) tract of a bilaterian is a “through-gut” system with oral and anal openings, accommodating the perennial objectives of prey intake, digestion, and discharge of remnants (Hartenstein and Martinez, 2019). As with other organs, the GI tract comprises populations of cells with varying degrees of specialization and proliferative potential. For instance, epithelial cells that line the luminal gut, including the esophagus, stomach, and small and large intestines, are constantly exposed to external stimuli and rapidly shed, having a short half-life of days during homeostasis (van der Flier and Clevers, 2009; Matsuo et al., 2017). These cells must be constantly replaced by somatic stem cells located at the base (esophagus, antral stomach, and intestines) or closer to the lumen (stomach corpus) (Barker, 2014). In contrast, differentiated cells in the GI tract that produce hormones and enzymes that aid in digestion can have half-lives as long as months (e.g., chief cells or parietal cells in the stomach, hepatocytes, or pancreas acinar cells) (Ireland et al., 2005; Mills and Shivdasani, 2011; Matsuo et al., 2017). The majority of the latter cell types maintain their colonies through autoduplication, and since few are lost, they rarely multiply (Desai et al., 2007; Malato et al., 2011; Aure et al., 2015; Brown et al., 2022). The coexistence of diverse cell types in the same GI compartment is an example of substantial cell-cell interaction playing a key role in maintaining organ homeostasis.

Just as any other organ that must interact with its external environment, GI organs suffer from chronic environmental insults including viral and bacterial infections, which trigger mutagenic responses that are at the heart of carcinogenesis. The most common cause of stomach cancer is the class I carcinogen *Helicobacter pylori* (*H.pylori*) bacterium, with Epstein-Barr virus-associated gastric cancers also responsible for a significant portion of cases (Tokunaga et al., 1993; Cancer, 1994). Additionally, infections with the hepatitis B and C viruses continue to be the leading worldwide cause of hepatocellular carcinoma (Di Bisceglie, 1997; Arbuthnot and Kew, 2001); while tobacco use, alcohol intake, chronic and acute pancreatitis, are associated with pancreatic cancer (Alsamarrai et al., 2014; Wei et al., 2016). Injury to GI organs frequently involves multiple cell types in large areas of the affected organ. In addition to the direct integration of a causative pathogen’s DNA into host cells (as seen in hepatitis B virus infections) (Edman et al., 1980; Shafritz et al., 1981; Tu et al., 2017), chronic injury-induced altered environments and damaged intracellular signaling pathways often result in a sustained cancer risk even after the causative pathogen has been eradicated (Hatakeyama, 2014). This is observed in metachronous gastric cancer formation after endoscopic or surgical resection (Graham, 2014; Cho et al., 2017; Choi et al., 2018), and sustained cancer risk in a proportion of cured hepatitis C patients (El-Serag et al., 2016; Luna-Cuadros et al., 2022).

Metaplasia is the transformation of one type of cell into another type of cell that did not exist in that tissue at homeostasis (Giroux and Rustgi, 2017). Metaplasias have long been recognized as a reliable indicator of chronic inflammation and increased cancer risk. A number of cell types in multiple organs exhibit metaplasias in response to injury, and the GI tract is no exception. The presence of goblet cells in the stomach, known as intestinal metaplasia, has long served as an indicator of longstanding *H. pylori* infection and an elevated risk of gastric cancer (Correa et al., 2010; González et al., 2013; Lee et al., 2022). Likewise, the presence of goblet cells and transition of squamous epithelium into columnar epithelium in the esophagus—known as Barrett’s esophagus—is also a precursor to esophageal cancer (Spechler and Goyal, 1986; Spechler, 2002). Metaplasias occur in secretory cells in the GI tract as well. In response to various injuries such as those from *H. pylori* infection, stomach chief cells undergo metaplasia resulting in expression of Trefoil factor 2 (TFF2) and the enzyme pepsinogen (Goldenring et al., 2010; Goldenring, 2018; Goldenring and Mills, 2022). Hence, this is named spasmolytic polypeptide-expressing metaplasia (SPEM). Acinar cells are another type of secretory cell in the pancreas that produces digesting enzymes. These cells undergo acinar-to-ductal metaplasia (ADM) that can ultimately progress to pancreatic ductal adenocarcinoma (PDAC) through the formation of pancreatic intraepithelial neoplasia (PanIN) (Kopp et al., 2012; Storz, 2017).

Therefore, examining metaplasia in differentiated cells may help to fill the large gap in our knowledge on how chronic inflammation contributes to the development of cancer. The question of how differentiated cells respond to injury and become metaplastic at the molecular level is currently being explored on numerous fronts. Secretory cells in the stomach corpus (chief cells) and pancreas (acinar cells) have arguably been the most thoroughly studied in this regard and are an ideal tool for examining how cells manage their energy and resources at the molecular level during injury and recovery (Willet et al., 2018). At homeostasis, these cells are characterized by disproportionate enrichment of secretory machinery (e.g., rough endoplasmic reticulum (ER), Golgi apparatus, and secretory vesicles) and quiescence. Injury leads to an increase in cell plasticity in terms of both proliferative capacity and lineage determination. Quiescent acinar cells in the pancreas re-enter the cell cycle after injury and are also known to become ductal-like cells or tuft cells (DelGiorno et al., 2020; Ma et al., 2022). Likewise, chief cells in the stomach corpus start to re-enter the cell cycle after injury and can turn into other types of cells in the same organ, such as parietal, neuroendocrine, or pit cells (Stange et al., 2013; Caldwell et al., 2022). In the small intestine, various precursor secretory cells might take on the function of crypt base columnar (CBC) stem cells following the ablation of CBCs (Jadhav et al., 2017; Yu et al., 2018; Murata et al., 2020). Hepatocytes can transdifferentiate to cholangiocytes and *vice versa* under extreme injury (Raven et al., 2017; Schaub et al., 2018). In fact, cell plasticity is evolutionarily conserved and is even more prevalent in basal metazoans, such as Porifera, where archaeocyte with totipotent stem cell properties can readily transdifferentiate to differentiated choanocyte or pinacocyte and *vice versa* (Sogabe et al., 2019).

Interestingly, the response of secretory cells to injury occurs through an evolutionarily conserved, stepwise process, which was recently termed paligenosis (Willet et al., 2018). Paligenosis consists of three sequential stages: 1) autophagic-lysosomal degradation of organelles, 2) induction of metaplastic/embryonic genes, and 3) re-entry into the cell cycle. Numerous molecular characterizations of genes governing paligenosis have been performed and are reviewed in detail (Brown et al., 2022). Understanding these cell-intrinsic changes that occur during paligenosis serves as a window to investigate how other differentiated cells in the GI tract respond to the injury, become metaplastic, regenerate, and may become carcinogenic during the process.

Despite our progress in understanding paligenosis, we still lack knowledge of a component that is crucial in determining cell fate during injury: the ribosome. The generation and maintenance of adequate numbers of ribosomes are essential for their primary function of translation. However, what is even more important, especially during catabolic states such as during injury, is proper regulation and production of the minimal number of ribosomes to survive. This is because ribosome biogenesis (RiBi) is an energy-intensive biological activity that consumes the majority of a cell’s energy (Mayer and Grummt, 2006). In fact, 35%–60% of total transcription in rapidly developing yeast cells is devoted to rRNA, while 50% of RNA polymerase II transcription and 90% of messenger RNA (mRNA) splicing are allocated to ribosomal proteins (Warner, 1999; Moss and Stefanovsky, 2002; Cavanaugh et al., 2004). Also, 80% of a proliferating eukaryotic cell’s work is devoted to the creation of protein components (Schmidt, 1999). Consequently, it is essential for the cell to tightly control the number of ribosomes by regulating RiBi, maintenance, and degradation of ribosomes, as well as the primary function of translation, according to the energy state of a cell.

This review focuses on ribosomal behaviors that have been understudied but are found in diverse cell types upon injury, and highlights how this understanding of ribosomes can inform how we view the plasticity of the digestive tract.

## 2 Life cycle of ribosomes at a glance

### 2.1 Ribosome biogenesis

**FIGURE 1:**
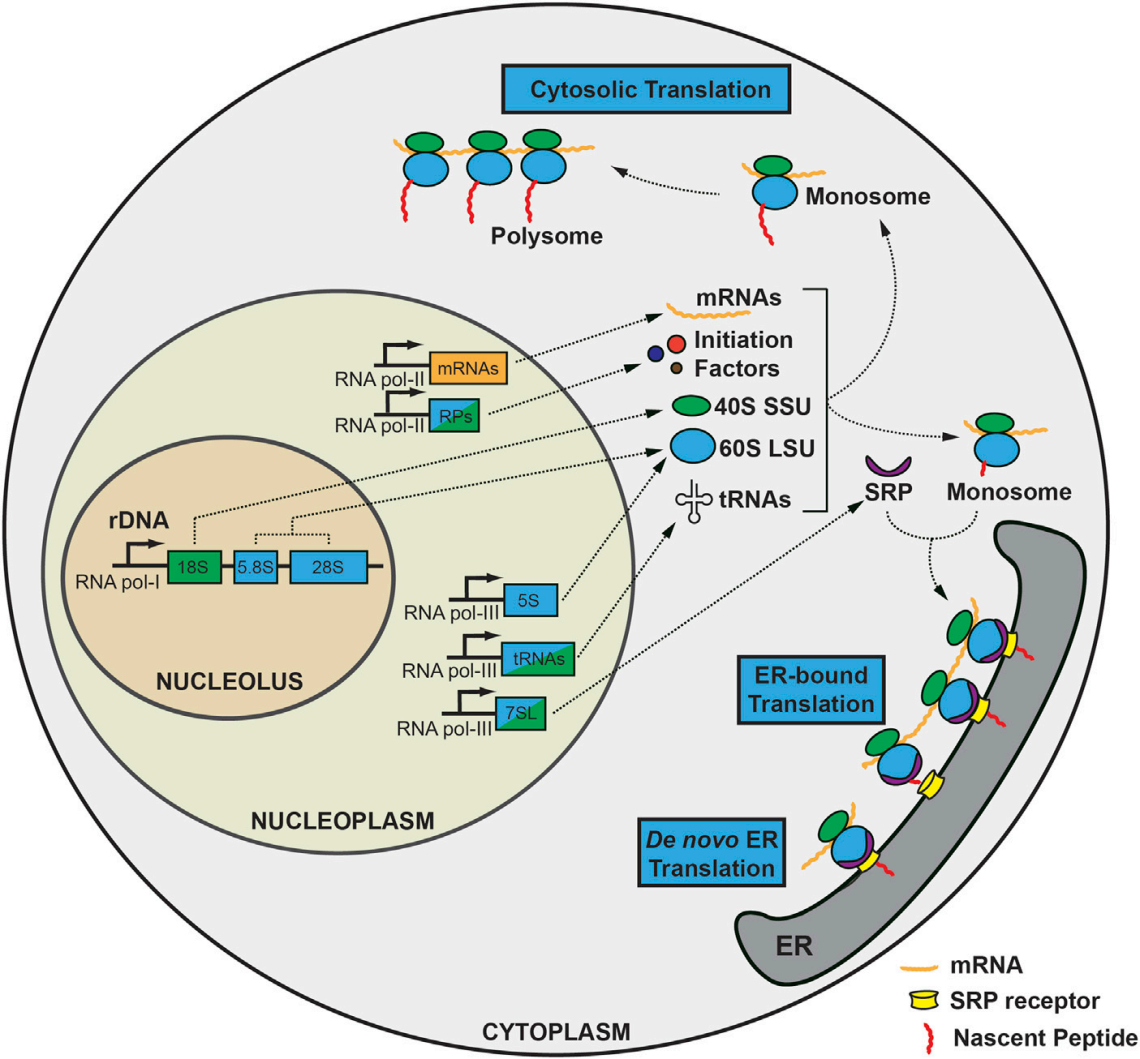
Overview of the biogenesis and translation of ribosomes during homeostasis. Ribosomal DNA (rDNA) transcription occurs in the nucleolus by RNA polymerase I. 18S rRNA is a component of the 40S small subunit (SSU), while 5.8S and 28S rRNA generates the 60S large subunit (LSU), along with 5S rRNA transcribed in the nucleoplasm. mRNAs and ribosomal proteins are transcribed by RNA polymerase II together with initiation factors and template mRNAs. In addition, RNA polymerase III generates tRNAs and 7SL-RNA in the nucleoplasm. SSUs undergo substantial modifications *en route* to the cytoplasm, where they encounter mRNA, initiation factors, and initiator methionine tRNA to form a preinitiation complex, followed by addition of 60S LSU to form 80S ribosome, also known as the monosome. The monosome can be translated in the cytoplasm as is or can form a polysome to generate nascent peptides more efficiently (cytosolic translation). On the other hand, mRNAs containing a signal sequence can translocate to the endoplasmic reticulum with the guidance of the signal recognition particle (SRP) complex as a monosome to produce secretory or membrane-bound peptides requiring extensive post-translational modification. Also, *de novo* translation initiation can occur on ER.

A ribosome is an evolutionarily conserved ribonucleoprotein (RNP) complex that is indispensable for translation (Petrov et al., 2015). Ribosomes exist in the cytoplasm or on the rough ER, where nascent polypeptides undergo extensive modification to be secreted or membrane-bound (Palade, 1955; Alberts et al., 2002) (Figure 1). *De novo* RiBi begins in the nucleolus, a well-known membrane-less structure in the nucleus. The nucleolus is composed of three sub-compartments: 1) the fibrillary center (FC), an electrodense region, bordered by 2) a dense fibrillary component (DFC), and 3) granular component (GC), where rRNAs mature and specific nucleolar proteins are localized (e.g., fibrillarin in the DFC, and nucleophosmin in the GC) (Leung and Lamond, 2003; Boisvert et al., 2007; Farley et al., 2015). The FC consists of nucleolar organizing regions (NORs), the number and location of which varies among species. For instance, in humans, five acrocentric chromosomes (the short arms of chromosomes 13, 14, 15, 21, and 22) contain NORs, whereas *Arabidopsis thaliana*, for instance, has two NORs on chromosomes 2 and 4, and *Mus musculus* has up to six (12, 15, 16, 17, 18, and 19) (Henderson et al., 1972; Dev et al., 1977; Haberer et al., 1996). Nevertheless, all NORs contain extensive tandem repeats of ribosomal DNAs (rDNAs), which may be required to accommodate a rapid increase in the demand for RiBi that is directly related to cell survival (Kobayashi et al., 1998). The transcription of RNA polymerase I and proteins such as UBTF1 and TCOF1 is essential for rDNA transcription (Grummt, 2003; Valdez et al., 2004; Sanij et al., 2008). In addition, nucleolar RNA polymerase II is directly engaged in stabilizing the nucleolus to ensure correct RiBi by producing R-loops, which are triplex nucleic acid structures, in addition to its role in small nucleolar RNA (snoRNA) transcription (Dieci et al., 2009; Abraham et al., 2020).

rRNAs are initially transcribed as long 47S (Svedverg Unit) pre-rRNA, that is cleaved in a regulated way on multiple spacer regions (5′- and 3′-external transcribed spacers, and internal transcribed spacers 1 and 2) to produce mature 18S, 28S, and 5.8S rRNA [reviewed in detail in (Henras et al., 2015)]. 5S rRNA is unique among backbone rRNA components in that it is transcribed using RNA polymerase III from tandem arrays of 5S rDNAs in the nucleoplasm (Ellis et al., 1988; Campell et al., 1992; Highett et al., 1993). It is later integrated alongside 5.8S and 28S rRNAs into the large subunit (LSU) of mature ribosomes with the help of two ribosomal proteins, RPL5 and RPL11, which regulate the positioning of 5S rRNA to the nucleolus (Steitz et al., 1988; Chakraborty et al., 2011; Ciganda and Williams, 2011; Sloan et al., 2013; Madru et al., 2015). Finally, chromatin modifiers such as methylation or acetylation of histones influence rDNA transcription, illustrating that initial rDNA transcription itself is a non-trivial, delicately coordinated process that involves all three RNA polymerases and massive energy consumption (Hirschler-Laszkiewicz et al., 2001; Santoro and Grummt, 2001; Mayer et al., 2006).

During the cleavage and maturation process of pre-rRNA, various post-transcriptional modifications occur, which involves three major processes: 1) hundreds of snoRNAs, the two major types of which are box C/D, which mediates 2′-O-methylation (with FBL, NOP56, and 58); and box H/ACA, which mediates pseudouridylation (with NHP2, NOP10, and DKC1), 2) 200 auxiliary proteins localized in different sub-compartments of the nucleolus (Henras et al., 1998; Newman et al., 2000; Dupuis-Sandoval et al., 2015; Massenet et al., 2017), and 3) acetylation at specific cytidine residues on 18S rRNA that is important for proper maturation of 18S rRNA, mediated by a single enzyme, NAT10 (Ito et al., 2014; Sharma et al., 2015; Sas-Chen et al., 2020). Through these processes, two subunits; the large subunit (LSU: 5S, 5.8S, 28S, and ribosomal large subunit proteins) and the small subunit (SSU: 18S and ribosomal small subunit proteins), are produced and are exported to the cytoplasm as subunits.

### 2.2 Translation at a glance

Translation of mRNA is the primary function of the ribosome (Aitken and Lorsch, 2012; Hinnebusch, 2014). The formation of a ternary complex in the cytosol initiates the translation process. With GTP as an energy source, the trimeric eIF2 complex can bind to Met-tRNA^iMet^, which then forms a 43S preinitiation complex with the 40S SSU and other initiation factors: eIF1, eIF1A, and eIF3. Additionally, mRNA is activated by the eIF4 complex (composed of eIF4E, eIF4G, a scaffold protein and RNA helicase, eIF4A), and poly(A)-binding protein (PABP), which aids in unwinding. The eIF4G scaffold protein allows for the formation of a “closed-loop” structure composed of eIF4F complex, mRNA, and PABP. Next, the 43S preinitiation complex loads onto the 7-methylguanosine cap structure at the 5′-proximal region of mRNA and begins scanning the 5′-untranslated region (UTR) to identify the start codon with the help of DHX29 (Pisarev et al., 2008; Abaeva et al., 2011; Hashem et al., 2013) until correctly placed at the ribosomal peptidyl-tRNA (P) site of 43S preinitiation complex. This is the point when eIF5 undergoes irreversible GTP hydrolysis on eIF2 to produce stable 48S preinitiation complex. Finally, as eIF1 and the eIF2-GDP complex are released and eIF5 is relocated, another GTPase, eIF5B, catalyzes the LSU to join the 48S preinitiation complex and form the 80S ribosome (Pestova et al., 2000). When eIF5B departs from the mature ribosome through GTP hydrolysis, only then does initiation begin. Eventually, the 60S LSU is introduced, and eIF5A is used to initiate elongation.

80S mature ribosomes can translate mRNA either as monosomes (one ribosome bound to one mRNA) or as polysomes (more than two ribosomes attached to a single mRNA), and in two distinct locations: as a free-floating form in the cytosol or on the rough ER. Thus, both cytosolic and ER-bound ribosomes are capable of existing as both monosomes and polysomes (Potter and Nicchitta, 2002; Ueno et al., 2012). Secretory or membrane-bound (endomembraneous) proteins associate with the ER as they are translated, while mRNAs encoding cytoplasmic proteins are translated on free ribosomes. This cellular compartmentalization is also known as mRNA partitioning (Blobel and Dobberstein, 1975; Palade, 1975). Co-localization to the ER is a co-translation process that needs the signal recognition particle (SRP) to stall and guide 80S monosomes carrying mRNAs with N-terminal signal sequences to the ER, where it binds to heterodimeric SRP receptors composed of SRPRA and SRPRB (Keenan et al., 2001). Although SRP is known to mediate the majority of translocation, SRP-independent pathways have also been implicated (Choi et al., 2000; Ast et al., 2013; Denic et al., 2013; Hermesh and Jansen, 2013).

Ribosomes have traditionally been thought to disassemble upon completion of translation. ABCE1 is involved in the disassembly of 80S ribosomes into subunits following canonical termination or ribosome quality control, and it has also been implicated in the “recycling” or re-initiation of 40S subunit synthesis (Nürenberg and Tampé, 2013). It has been thought that ribosomes not only disassemble but also dissociate from ER after translation termination (Mechler and Vassalli, 1975). However, this assumption has since been contested, at least during injury, when monosomes from fragmentation of polysomes after unfolded protein response were still connected to ER and these ER-bound ribosomes were able to translate after injury (Stephens et al., 2005).

Ribosomal components are degraded when they are improperly constructed, not utilized, or when translation goes awry. rRNAs undergo non-functional rRNA decay (NRD) via multiple pathways, including the TRAMP-exosome pathway, 18S NRD, and 25S NRD, as others have reviewed in detail (Lafontaine, 2010). It is intriguing that the 18S NRD, which occurs during SSU formation, is intimately associated with the no-go decay of mRNA and P-body localization, while the ubiquitin-proteasome system is responsible for the degradation of LSUs (Cole et al., 2009). Ribosomal proteins are monitored and regulated as well. Unless the endogenous RPL26 pool is depleted, overexpression of RPL26 in yeast does not result in its integration into the ribosome or accumulation of RPL26, as it is continuously degraded via the ubiquitin-proteasome pathway (Sung et al., 2016).

### 2.3 Factors influencing the quantity and quality (or function) of ribosomes

Cells must continuously monitor and maintain the optimal number of ribosomes based on their needs (translation) and costs (energy expenditure). This balance is maintained by multiple “sensors” and “executors,” which include most of the key signaling pathways that maintain this equilibrium. In this section, we review how mTORC1, Myc, and AMPK play a significant role in the regulation of RiBi, although it should first be emphasized that no factor is solely responsible for governing or the regulation of the RiBi pathway.

mTORC1 is a well-known factor for sensing nutrients and is also linked to cellular growth and proliferation in various species, including multiple phyla of animals (Mamane et al., 2006), plants (Chen et al., 2018), and yeast (De Virgilio and Loewith, 2006; Chan, 2009). mTORC1 is positively affected by “anabolic signals,” such as growth signals, mitogens, and nutrient availability, which are mediated through PI3K, AKT, and TSC1/2 (Fingar et al., 2002; Hirose et al., 2014). mTORC1 mediates downstream signaling through phosphorylation of ribosomal protein S6 kinases, S6K1 and S6K2, and through phosphorylation of RPS6 and eukaryotic initiation factor 4E-binding protein 1 (4E-BP1) (Shima et al., 1998; Pende et al., 2000; Pende et al., 2004). Not surprisingly, mTORC1 regulates both translation and RiBi (Jastrzebski et al., 2007; Iadevaia et al., 2014). To ramp up RiBi, mTORC1 can directly affect: 1) RNA polymerase I transcription, 2) translation of ribosomal proteins via the 5′- terminal oligopyrimidine (TOP) motif, and 3) increasing RNA polymerase III transcription.

RNA polymerase I activity is regulated directly by mTORC1 through various mechanisms including modulation of TIF-IA activity or influencing the Ccr4-Not complex, which can be inhibited by a specific mTORC1 inhibitor, rapamycin (Mayer et al., 2004; Laribee et al., 2015). Rapamycin can also inhibit the processing of pre-rRNAs at the conversion of 30S to 21S and the processing of 32S rRNA (Iadevaia et al., 2012), suggesting that mTORC1 regulates overall rRNA transcription and processing at multiple points.

mTORC1 also influences RNA polymerase II-transcribed proteins involved in RiBi. It was demonstrated that properly functioning S6K1 and S6K2 are required for transcription of RiBi-related factors (e.g., NOP56, NOP14, and GAR1) in the liver of mice refed after fasting (Chauvin et al., 2014). Additionally, translation of the proteins with 5′ TOP motifs, a sequence of nucleotides that begins with 5′-cytidine, followed by tandem pyrimidines up to 15 (Yoshihama et al., 2002), is influenced by mTORC1 through 4E-BP1 (Iadevaia et al., 2008; Thoreen et al., 2012; Miloslavski et al., 2014). The motif is found in the promoter region of most ribosomal proteins and translation machinery components, including initiation factors (e.g., eIF3E, eIF3F, and eIF3H) and elongation factors such as eEF1A and eEF2 (Iadevaia et al., 2008; Cockman et al., 2020).

Finally, mTORC1 influences RNA polymerase III. In yeast, Tor1 binds to 5S rDNA chromatin and is involved in 5S rRNA production (Wei et al., 2009). RNA polymerase III is bound to and transcriptionally regulated by Maf1, the function of which is inhibited by phosphorylation on its Ser-60, 68, and 75 sites by mTORC1 (Kantidakis et al., 2010; Michels et al., 2010; Shor et al., 2010).

Myc is a transcription factor that functions as the “master regulator” of global translation and cell cycle control. Therefore, it is not surprising that Myc regulates the expression of genes involved in the synthesis of rRNA and ribosomal proteins (van Riggelen et al., 2010). c-Myc is required for the direct conversion of 47S rRNA precursors into mature 18S and 28S rRNA (Schlosser et al., 2003; Arabi et al., 2005; Grandori et al., 2005), and production of proteins involved in RiBi such as nucleolin, dyskeratin, or fibrillarin (Coller et al., 2000; Alawi and Lee, 2007), as well as ribosomal proteins specifically (Kim et al., 2000). In addition, numerous c-Myc transcriptional targets are translation initiation factors, such as eIF4E (Schmidt, 2004). Lastly, c-Myc directly triggers the transcription of RNA-polymerase III (Gomez-Roman et al., 2003).

AMP-activated protein kinase (AMPK) is another critical cellular energy sensor that regulates diverse metabolic processes under stress and can also have an effect on RiBi (Hardie, 2011; Hardie et al., 2012). Specifically, when activated by an increase in the AMP/ATP ratio, such as in times of energy shortage, AMPK can phosphorylate a single serine residue (Ser-635) on the RNA polymerase I-associated transcription factor TIF-IA, thereby inhibiting its binding to SL-1 and preventing the assembly of the TF complex (Hoppe et al., 2009).

## 3 Ribosomal behavior during stress

**FIGURE 2:**
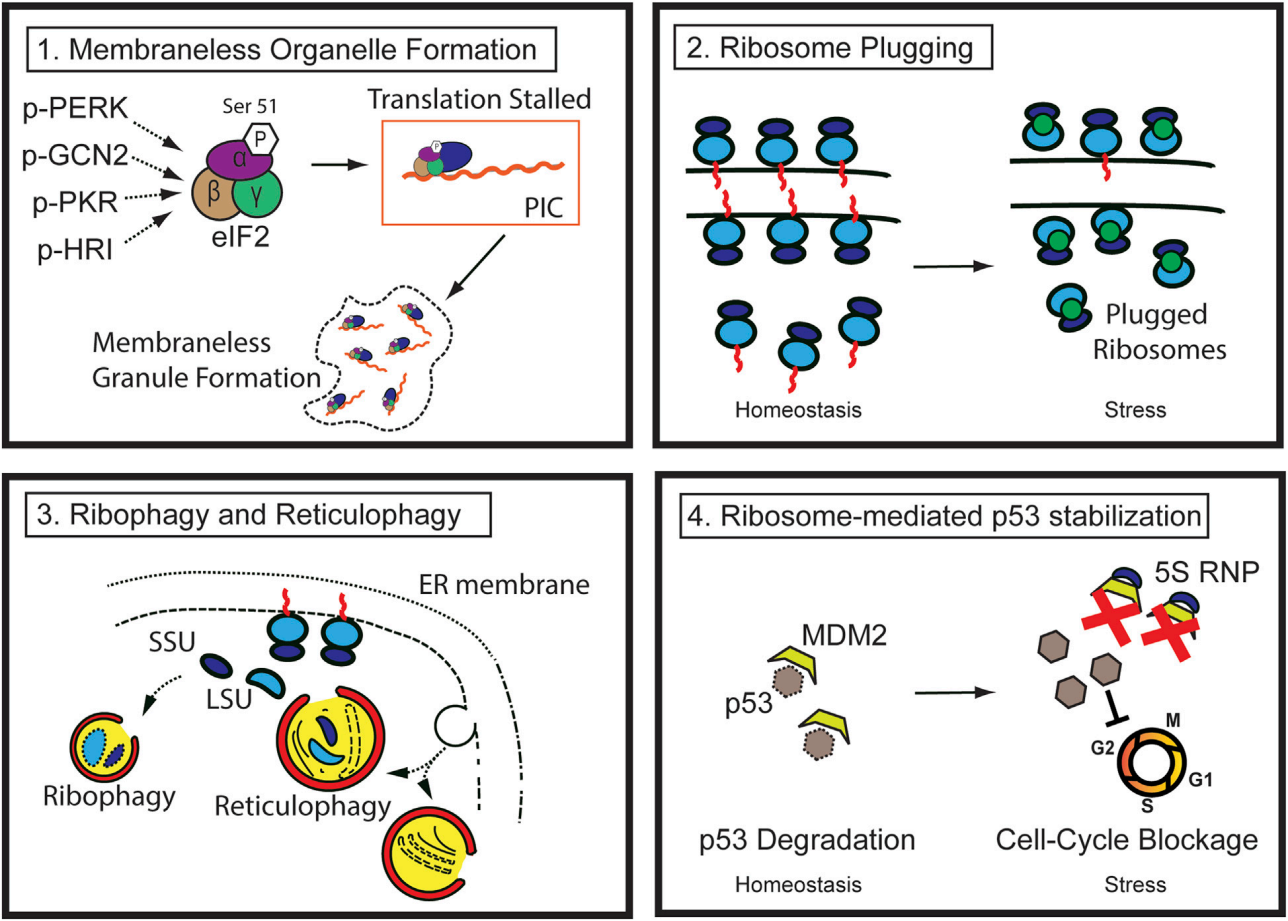
Pathways of ribosome regulation during injury (1) During stress, multiple kinases phosphorylate the Serine 51 residue of eIF2 alpha, reducing translation efficiency. The stalled preinitiation complex can be localized to membraneless granules, such as stress granules. (2) Ribosome degradation can be prevented by ribosome-plugging proteins (green circles), the majority of which are evolutionarily conserved. (3) Ribosomes in the cytosol and those attached to the rough ER are susceptible to autophagic destruction through ribophagy and reticulophagy. (4) Ribosomal proteins (such as RPL5 and 11) and 5S ribosomal RNA complex (5S RNP) from degraded ribosomes or other errors in ribosome biogenesis stabilize p53 by sequestering and inhibiting MDM2, ultimately blocking cell cycle progression.

The cell survival program is triggered at both the cellular and tissue levels in response to stress. So far, it is evident that tissue response to injury cannot be explained by a “one-size-fits-all” theory, as it involves drastic structural and biochemical changes both at the cell and tissue level, including dynamic alteration in proliferative potential, remodeling and removal of organelles, cell death through various routes, and eventual cellular and tissue regeneration. A number of cellular phenotypes such as autophagy, stress granule formation, P-body formation, or translational blockage have aided in our understanding of how cells can cope with injury and regenerate, but each can explain only a part of the whole process, necessitating a comprehensive understanding of the process. Here, we propose that the ribosomes and the ER are at the center of all morphologic changes and fate determination processes after injury and that understanding how ribosomes are handled is crucial to understanding cell behavior during injury. Dynamic regulation of both the quantity and the function of ribosomes is crucial for successful regeneration. Thus, the canonical role of ribosomes must be reconsidered, particularly under stress, since it is becoming clear that ribosomes are not static entities that perform only one job—translation—but are instead dynamic, versatile, and crucial players in the regeneration process (Figure 2).

Specifically, the initial response of injured cells is to decrease the biogenesis of the nascent ribosomes and shut down translation to lessen the workload and consumption of energy used by these processes. Rough ER also experiences functional or structural changes. Existing cytosolic and rough ER ribosomes undergo autophagy (also known as ribophagy and reticulophagy, respectively). Furthermore, perturbation of RiBi and disruption of ribosome homeostasis result in p53 stabilization in a manner similar to that of ribosomopathy, which will be discussed in detail in the following sections.

### 3.1 Global synthesis block, selective translation, and membraneless organelle formation

Global translational block occurs in response to a variety of stimuli, including ER stress (Ron, 2002), iron deficiency (Han et al., 2001), amino acid starvation (Dever et al., 1992), infection (Chakrabarti et al., 2012), and hypoxia (Staudacher et al., 2015). This makes sense from a metabolic standpoint, as ribosome synthesis and translation are extremely energy-intensive processes. Moreover, this would help reduce the net flux of nascent polypeptides from the cytosol to the ER, which is required when misfolded proteins accumulate during ER stress. Consequently, cells have evolved delicate mechanisms to inhibit translation via multiple processes. This occurs via three mechanisms: 1) reduction in the total number of ribosomes by inhibition of RiBi, 2) reduction in translation efficiency, and 3) reduction in the number of functional ribosomes.

Reducing the total number of ribosomes by inhibiting the transcription of the genes involved in RiBi and ribosomal protein genes can be a rather rapid and straightforward process, which is noted in response to various types of stress in yeast (Warner, 1999; Gasch et al., 2000; De Nadal et al., 2011). Also, stressors are often linked to the rapid shutdown of RNA polymerase I that is triggered by the DNA-damage response, mediated by ATM kinase activity and the repair factor proteins NBS1 and MDC1 (Kruhlak et al., 2007). Moreover, promoter-bound RNA polymerase II is susceptible to targeted degradation (Steurer et al., 2022). In addition, it is widely known that stress inhibits RNA polymerase III (Marshall et al., 2012; Rideout et al., 2012; Moir and Willis, 2013). These mechanisms reduce the net number of nascent ribosomes and, by extension, the ribosome pool.

RiBi is further blocked at the processing step. In response to stress, such as the integrated stress response, pre-rRNA processing ceases at a relatively early stage of A'/01 processing (Szaflarski et al., 2022). Moreover, suppression of mTORC1, which is known to occur during injury across multiple phyla (Di Cara et al., 2018; Willet et al., 2018), can reduce phosphorylation of 4E-BP1, which strengthens its association with eIF4E and interferes with its interaction with eIF4G1, thereby limiting translation initiation, particularly for transcripts containing 5′-TOP motifs (Gingras et al., 1999; Thoreen et al., 2012). Unphosphorylated LARP1 is an RNA-binding protein that interacts with the 5′- and 3′- UTR of ribosomal protein mRNAs. mTORC1 and Akt have been shown to phosphorylate LARP1 and promote translation of the ribosomal proteins (Hong et al., 2017). Thus, during stress, a decrease in mTORC1 can result in a decrease in ribosomal protein levels and a subsequent decrease in the number of ribosomes.

Another well-known global suppression occurs directly at the translation level. Global synthesis blocking involves stalling or blocking at the initiation step—a well-known rate-limiting step of translation (Richter and Coller, 2015). The most well-known mechanism for stress-induced blocking is serine 51 phosphorylation on the alpha subunit of eIF2, mediated by four kinases, GCN2, PKR, PERK, and HRI, which arrests translation by leading to a considerable decrease in protein synthesis efficiency because it changes the affinity for binding eIF2B, a guanidine exchange factor that re-charges eIF2 with GTP and is critical to initiating another round of translation (Taniuchi et al., 2016).

Frequently, stalled 48S preinitiation complexes are retained in newly formed membraneless granules. The stress granule is the best-known example of such membraneless organelles that can emerge in response to stressors such as those in the integrated stress response (Kedersha et al., 1999; Scheuner et al., 2001; McEwen et al., 2005). The assembly and disassembly of stress granule is a dynamic process induced by stress and ceases when stress is relieved (Wheeler et al., 2016). In this regard, the assembly of stress granules may serve as a hub for the storage of stalled mRNAs and translation factors during stress until they can be released back to restore homeostasis, conserving energy during the catabolic phase of injury and enabling the cell to preserve selective mRNPs from the translation pool that may be essential for the recovery phase (Buchan and Parker, 2009; Wheeler et al., 2016).

It should be noted, however, that not all translation is blocked. In fact, ATF4, IFRD1, and PPP1R15B are among the few transcripts that are preferentially translated during a global translational block. Mechanistically, upstream open reading frame (uORF) translation is responsible for preferential translation of most of these transcripts (Vattem and Wek, 2004; Zhao et al., 2010; Andreev et al., 2015), and these transcripts play a variety of roles in the stress response, suggesting that cells have implemented a sophisticated machinery for their survival during stress. Translation on ER during injury also deserves attention. Clearly, *de novo* translation initiation can occur on the ER (Jagannathan et al., 2014), where both monosomes and polysomes can form (Voigt et al., 2017). During ER stress, phosphorylation of PERK, a critical kinase residing on the ER membrane, can directly sense and phosphorylate the eIF2α that results in cytosolic translational block. However, translation on the ER continues to occur and stress response transcripts such as *Atf4* can be translated on the ER, thus serving as a “safe haven” during injury. On the other hand, there are injuries that are detrimental to the ER, such as the cerulein injury model in pancreatitis. In this situation, since the ER platform is no longer present, ribosomes may be degraded along with the ER or need to disengage from the ER to initiate a new round of translation in the cytosol. These findings suggest that regulation of the endoplasmic reticulum (ER) during stress can have a significant but non-uniform effect on the cell during stress and regeneration, an aspect that warrants further investigation.

In short, translation is generally impeded during injury, however it should be noted that cells are constantly sensing their nutritional and energy state and preparing for regeneration during the anabolic phase.

### 3.2 Ribosome plugging

Translational efficiency can be regulated by modulating the number of existing, functional ribosomes. The concept of impairing ribosome function without destruction is best studied in prokaryotes. Bacteria are known to produce “hibernating” ribosomes by forming 100S dimers (70S + 70S) during injury (Ortiz et al., 2010; Polikanov et al., 2012; Basu and Yap, 2017; Beckert et al., 2017; Matzov et al., 2017; Beckert et al., 2018). Ribosome modulation factor, hibernation promoting factor, and ribosome associated inhibitor occupy the decoding center, mRNA binding channel, and acceptor (A) and peptidyl (P) sites within the ribosomes (Yoshida et al., 2002; Ueta et al., 2005; Ueta et al., 2008). This mechanism is evolutionarily conserved in eukaryotes. Arguably, Stm1 in yeast is the best studied protein of this kind that is activated in response to diverse stimuli (e.g., glucose starvation) and binds and protects ribosomes from proteasomal degradation (Van Dyke et al., 2004; Van Dyke et al., 2009; Ben-Shem et al., 2011; Brown et al., 2018; Shetty et al., 2023). The structure and function of Stm1 is evolutionarily conserved in mammals as an ortholog, SERPINE mRNA binding protein 1 (SERBP1), although it is unclear if it is functional during injury (Brown et al., 2018). IFRD2 is another ribosome plugging protein, which was structurally found to occupy the P and E sites of the ribosome, making it incompatible with translation in rabbit reticulocytes (Brown et al., 2018). The presence of these ribosome plugging proteins seems to be restricted to a few organs, and the organ-specific function of these proteins at homeostasis is relatively vague (Brown et al., 2018; Hopes et al., 2022). Recently, it was shown that suppression of mTORC1 stimulates Stm1/SERBP1 activity, offering a clue on the mechanism by which a perturbation of homeostasis may affect ribosome regulation (Shetty et al., 2023). Interestingly, it was shown that IFRD1, a paralog of IFRD2 that shares high degree of structural similarity, increases after various types of stress *in vitro* and *in vivo* (Ndum, 2010; Zhao et al., 2010; Miao et al., 2020), which may provide an important clue to explain how ribosome plugging can affect tissue regeneration. Not surprisingly, evolutionarily conserved declamping elements also exist, which play a role in the release of monosomes from “plugged” ribosomes, allowing them to engage in translation and polysome formation following the cessation of stress. The best-known example is Dom34-Hbs1 in yeast, whose orthologs in mammals are PELOTA and HBS1L (Van Den Elzen et al., 2014). The yeast late-annotated short open reading frame 2 (*Lso2*) is another declamping factor involved in translational recovery following malnutrition during stationary phase with a comparable function to its mammalian counterpart, a coiled-coil domain containing short open reading frame 124 (CCDC124) (Wells et al., 2020). In short, ribosome plugging and de-plugging may be an evolutionarily conserved mechanism that performs a multifunctional role at homeostasis and during injury by inhibiting translation, and conserving energy during the catabolic phase.

### 3.3 Ribophagy and reticulophagy

Reducing the absolute amount of existing machinery for translation (i.e., ribosomes) is another strategy implemented by cells to reduce the translation workload and energy expenditure. As ribosomes exist in a free-floating form in the cytosol or are attached to the ER, their degradation can occur through two distinct pathways: direct degradation of ribosomes by autophagosomes (ribophagy) and in conjunction with autophagy of rough ER (reticulophagy). Although ribophagy has been documented for decades with transmission electron microscopy (Eskelinen et al., 2011), only recently have its receptors and precise mechanism been defined. In yeast, nitrogen deficiency induces selective ribophagy via the Ubp3p/Bre5p ubiquitin protease complex (Kraft et al., 2008). It is intriguing that the human orthologs of Bre5p are the proteins G3BP1 and G3BP2, the former of which is involved in the production of stress granules (Cohen et al., 2003), suggesting a possible link between these well-known contingency methods that cells utilize during stress. In addition, fasting or mTORC1 suppression induces selective ribophagy, where a ribosome-binding protein, NUFIP1, can interact with both ribosomes and LC3B, thereby serving as a specialized receptor for ribophagy (Wyant et al., 2018).

In contrast, reticulophagy is an autophagic process that selectively degrades the ER (Bernales et al., 2006; Schuck et al., 2009). Reticulophagy is essential for maintaining proteostasis during homeostasis and during injury by degrading misfolded proteins and preventing the unfolded protein response (Meusser et al., 2005; Römisch, 2005). It has been demonstrated that phagophores can form near the ER and are physically associated with the ER membrane (Hayashi-Nishino et al., 2009; Ylä-Anttila et al., 2009). There are a few known reticulophagy receptors. Starvation stimulates the reticulon-like proteins ATG40 and FAM134B, which are responsible for ER fragmentation and autophagosome targeting. To remove misfolded proteins, FAM134B interacts with calnexin, an ER-resident lectin chaperone, and the autophagosome membrane-associated protein LC3 (Forrester et al., 2019). Hypoxia also stimulates reticulophagy by forming a complex between FAM134B and the ER chaperone BiP (Chipurupalli et al., 2022). CCPG1 is another reticulophagy-related protein that has recently emerged as an important non-canonical cargo receptor that facilitates reticulophagy by binding directly to core autophagic proteins via the LC3-interacting region or FIP200 as well as ER luminal proteins such as prolyl 3-hydroxylase family member 4 (P3H4) (Smith et al., 2018; Ishii et al., 2023). Insufficiency of CCPG1-mediated ER proteostasis in hypomorphic mice led to excessive ER stress and pancreatic tissue damage (Smith et al., 2018). Although research on reticulophagy that focuses on the destruction of ribosomes is very limited, it is clear that ribosomes attached to the ER membrane for translation are highly likely to be destroyed as bystanders, while the degradation of the ER membrane can serve as a source for autophagosomes.

In short, ribophagy and reticulophagy are essential processes that enable cells to adapt to environmental stresses and preserve cellular homeostasis, which is essential for the injury response.

### 3.4 Ribosome-mediated p53 stabilization during injury

Another way ribosomes are involved in the cell’s injury response is by serving as crucial mediators of the p53 stabilization. Mutations or reduced expression in ribosomal proteins, or factors involved in RiBi, have been known to cause a variety of developmental defects known as ribosomopathies that are associated with p53 stabilization. This provides evidence for the close relationship between ribosomes and p53 stability (Narla and Ebert, 2010; Fumagalli and Thomas, 2011; Farley-Barnes et al., 2019). For example, Diamond-Blackfann anemia that occurs with mutations in genes coding for ribosomal proteins such as RPS19, RPS24, RPL5, or RPL11, that result in hypoplastic, macrocytic anemia and an elevated risk of cancers such as acute myelocytic leukemia, can at least partially be corrected by deletion of p53 (Draptchinskaia et al., 1999; Willig et al., 1999; Gazda et al., 2004; Gazda et al., 2006; Gazda et al., 2008; Boria et al., 2010; Vlachos et al., 2012; Bhoopalan et al., 2023). Treacher-Collins syndrome, another ribosomopathy caused by autosomal dominant mutations of the *Tcof1* gene, leads to a deficiency in ribosome synthesis and a p53-dependent apoptosis in neural crest cells of embryos, leading to craniofacial birth defects (Sakai and Trainor, 2009; Trainor et al., 2009), which can be rescued by knocking out p53 (Jones et al., 2008).

Although best studied in congenital defects related to ribosomopathies, the p53 sensing of RiBi also occurs in non-mutated, “normal” cells; however, it appears that this process is particularly important during injury when RiBi is inhibited, or ribosome integrity is compromised. Perhaps, the best-known example of this phenomenon is the formation of the 5S RNP complex, composed of 5S rRNA, RPL5, and RPL11. The 5S rRNP complex may translocate from the cytoplasm (where the vast majority of mature ribosomes are) to the nucleoplasm, and bind and sequester MDM2 (Sloan et al., 2013). It is also possible that a “nascent” 5S RNP complex generated in the nucleoplasm sequesters MDM2 directly during stress (Donati et al., 2013). Furthermore, nucleolar proteins or even the nucleolus itself can serve as a “stress sensor” whose disruption results in the stability of p53 (Rubbi and Milner, 2003). NPM1, a nucleolar protein found in the granular center, binds to HDM2 and functions as a negative regulator of the p53-HDM2 interaction (Kurki et al., 2004). Also, nucleolin translocates from the nucleolus to the nucleoplasm under stress and directly interacts with p53 (Daniely et al., 2002). A partial hepatectomy in mice deficient in RPS6, a component of 40S SSU, resulted in failure of hepatocytes to undergo cell cycle progression at G1-S phase. This is the phase during which p53 serves as a key checkpoint, demonstrating a close relationship between ribosome integrity, p53, and proliferation after injury *in vivo* (Volarevic et al., 2000). This extends to the intriguing idea that the ribosome may be the hidden key linker between perturbation of major signaling pathways, such as mTORC1 or Myc, and cell-cycle progression/p53 stabilization, all of which are key processes that occur during injury (Willet et al., 2018), allowing ribosomes to be targeted for the facilitation or modulation of the injury process.

## 4 Conclusion

Here, we have shown that ribosomes are regulated in a distinct and specific manner during injury that is crucial for cells to cope with injury and regenerate. Our new understanding will transform ribosomes from passive translational machinery to an essential injury responder and cell fate determinant. This will allow individual observations that have been made in injured cells (e.g., stress granule formation, translational block, ribosome plugging, autophagy, p53 stabilization, cell death, and cell cycle reentry) to be comprehended as a whole, with the ribosome and the ER at the center. Yet, many questions remain unanswered. For instance, we do not know whether the number and function of ribosomes differ significantly by cell type and how this would influence the mode of ribosome regulation during injury. In addition, further studies may reveal how the remodeling or destruction of the ribosomes and the ER during injury alters the translational profiles of ribosomes. Addressing unanswered questions such as these will aid in expanding our understanding of tumorigenesis and the regeneration of multiple types of cells in diverse tissues.
